# A Rare Case of Kissing Gastric Ulcers Secondary to Non-steroidal Anti-inflammatory Drug (NSAID) Intake

**DOI:** 10.7759/cureus.27490

**Published:** 2022-07-30

**Authors:** Sri Hari Priya Vemulakonda, Souradeep Dutta, Ankit Jain, Abhinaya Reddy, Vishnu Prasad Nelamangala Ramakrishnaiah

**Affiliations:** 1 Surgery, Jawaharlal Institute of Postgraduate Medical Education and Research, Puducherry, IND

**Keywords:** endoscopy, analgesic, nsaid induced gastritis, gastric ulcer, kissing ulcer

## Abstract

Peptic ulcer disease is a heterogeneous disease caused by the imbalance between mucosal protective and aggressive factors. Such ulcers are common in the anterior wall of the duodenum and gastric antrum. Kissing ulcers, although commonly reported in the duodenum, are rarely seen in the stomach. We present a rare case of an 85-year-old lady who had an index presentation of sudden onset hematemesis following ibuprofen intake. Endoscopy revealed kissing gastric ulcers, which are extremely rare secondary to non-steroidal anti-inflammatory drugs. She had complete healing after treatment with proton pump inhibitors.

## Introduction

A peptic ulcer is defined as a gastric or duodenal mucosa erosion that extends through the muscularis mucosa. *Helicobacter pylori*-associated gastritis and ingestion of nonsteroidal anti-inflammatory drugs (NSAIDs) are the two most common causes of peptic ulcers [1]. Other common causes are smoking, stress, foreign body, caffeine intake, and trauma [1,2]. Kissing ulcers are a pair of ulcers facing each other on the opposite walls of the stomach or duodenum [2]. Although common in the duodenum, kissing ulcers in the stomach have been rarely reported in the literature. We report a rare case of kissing gastric ulcers secondary to ibuprofen (NSAID) intake.

## Case presentation

An 85-year-old lady presented to the emergency with new-onset hematemesis for one day. She had three episodes of hematemesis with no history of melena. She was a known hypertensive for 20 years, which was well controlled on once daily amlodipine. She also had age-related osteoarthritis, which aggravated in the last week, and she was on twice daily ibuprofen tablets (over the counter) for the past five days. She had no history of jaundice, smoking, alcohol abuse, or trauma. There was no past history of NSAID analgesic use.

**Figure 1:**
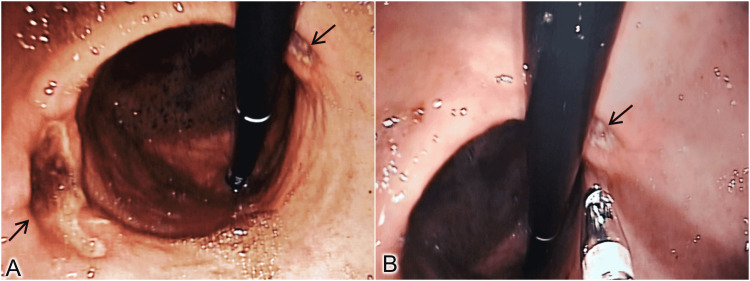
Upper gastrointestinal endoscopy images. A - Kissing ulcers in the stomach (Black arrows) seen during J maneuver, B - Biopsy being taken from the ulcer site.

At presentation, her hemodynamics were stable. Her physical examination was normal. Upper gastrointestinal endoscopy revealed two ulcers in the mid-body of the stomach, on the anterior and posterior walls, facing each other. They were of size 3x2 cm and 1x2 cm, respectively, with sloughed base and no active bleed, surrounded by normal gastric mucosa (Figure 1A). The ulcer on the anterior gastric wall was of Forrest class IIc, and the one on posterior wall was Forrest class III. The rapid urease test from gastric mucosa was negative. Biopsies sampled from both the ulcers were negative for malignancy and *H. pylori* (Figure 1B). NSAID intake being the only risk factor identified, they were classified as Johnson Type V ulcers. 

**Figure 2:**
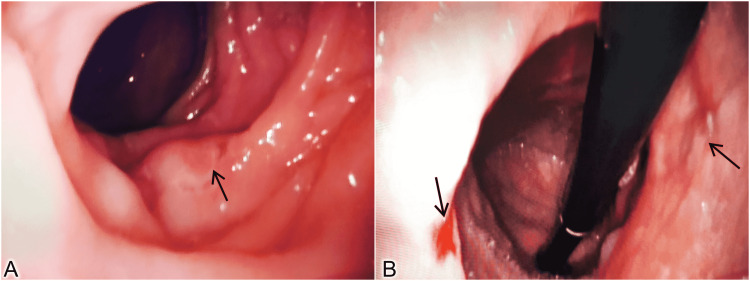
Post treatment upper gastrointestinal endoscopy images showing resolving ulcers. A – Black arrow showing healed ulcer site, B – Photo taken during J maneuver of both the healing ulcer sites

She was advised to stop ibuprofen and was conservatively managed with oral proton pump inhibitors. Repeat endoscopy after one month showed healing ulcers with surrounding normal mucosa (Figure 2).

## Discussion

Peptic ulcer disease (PUD) is a heterogeneous disease caused by the imbalance between mucosal protective factors like mucosal bicarbonate secretion, blood flow, cell renewal, prostaglandin production, and aggressive factors like *H. pylori* infection, NSAID use, smoking, alcohol abuse, stress, and trauma. Such ulcers are common in the esophagus, stomach, and duodenum. Among all PUD, 10-20% present with complications such as perforation and gastric outlet obstruction, the most common being upper gastrointestinal bleed [3].

Kissing ulcers are a pair of ulcers present on opposite walls in the stomach or duodenum [2]. Although commonly reported in the duodenum (1.5%) [1,2], kissing ulcers in the stomach are rarely reported in the literature. On our extensive search, we could find only four such case reports [2,4-6]. Out of these, two were due to trauma [2,5], and the other was due to a percutaneous endoscopic gastrostomy tube [6]. The etiology in the fourth case was not mentioned; however, the use of an NSAID was ruled out [4]. 

NSAID analgesic use is associated with many gastrointestinal problems, leading to significant morbidity and even death. The prevalence of peptic ulcers in NSAID users is 14-25% and is usually gastric more than duodenal. However, up to 50% of the endoscopic proven gastric ulcers have an association with NSAID analgesics [7]. Moreover, NSAID consumption in regular doses, even for a short period, increases the probability of PUD [3]. Other risk factors that can increase the severity of NSAID impact include advanced age (>70 years), previous ulcer history, the first three months of treatment with NSAID analgesic intake, smoking, other cardiovascular comorbidities, *H. pylori*, and use of corticosteroids or anticoagulants [7].

Continuation of NSAID analgesics in a proven case of gastric ulcers retards their healing. Therefore the first step toward treatment is discontinuing the analgesic drug or reducing the dose if the cessation is not feasible. However, if cessation or reduction of the dose of NSAID analgesics is not feasible, using proton pump inhibitors or histamine type-2 receptor antagonists along with an NSAID can reduce ulcer incidence [8]. The use of cyclooxygenase-2 specific NSAID analgesics is also recommended as an option. Rarely surgical intervention is required in acute presentations such as intractable ulcer bleed and perforation [9].

## Conclusions

Although reported a few times in the duodenum, kissing ulcers are rarely reported in the stomach. Although the precise pathophysiology is still mostly unknown, this unusual condition may be caused by abrupt abdominal trauma or a bout of acute NSAID ingestion. Cessation of NSAID analgesic use and the addition of proton pump inhibitors leads to complete healing.
